# Bleaching Teeth

**Published:** 1887-11

**Authors:** 


### BLEACHING TEETH.

At times there seems to be exhibited either a lack of comprehension of the principles involved in the bleaching of teeth, or speakers in dental meetings do not express themselves clearly. In the report of the discussions at the International Congress, for instance, some of the speakers refer to chlorine gas as the bleaching agent. There is no power in chlorine to discharge color. The active agent is oxygen. When chlorine is used in the process, water is absolutely essential, and hence the instructions sometimes given to exercise care in keeping the cavity into which the chlorine gas is admitted dry, show an ignorance of the principles involved. Moisture is essential, for the chlorine, having a great affinity for the hydrogen of the water, unites with it and sets free the oxygen, and this is the agent which does the work.

The decomposition of common salt in a cavity, by electrolysis or any other means, sets free the chlorine, which unites with the hydrogen of any water that may be present, and in turn liberates the oxygen, and this unites with the coloring matter, usually some form of carbon, and discharges its color.

When Labarraque's solution (chlorinated soda) is used in connection with alum (sulphate of aluminum and ammonium), the process is essentially the same, chlorine being set free and uniting with the hydrogen of water that may be present, and liberating the oxygen. Chlorine gas is commonly used in bleaching teeth because it is the most easily generated of the agents which will liberate free oxygen.

